# Effects of blood-flow restricted exercise versus conventional resistance training in musculoskeletal disorders—a systematic review and meta-analysis

**DOI:** 10.1186/s13102-023-00750-z

**Published:** 2023-10-25

**Authors:** Stian Langgård Jørgensen, Signe Kierkegaard-Brøchner, Marie Bagger Bohn, Mathias Høgsholt, Per Aagaard, Inger Mechlenburg

**Affiliations:** 1 Department of Occupational and Physical Therapy, Horsens Regional Hospital, Horsens, Denmark; 2 H-HIP, Department of Occupational and Physical Therapy and Department of Orthopedic Surgery, Horsens Regional Hospital, Horsens, Denmark; 3 Department of Clinical Medicine, Aarhus University, Aarhus, Denmark; 4 Department of Sports Science and Clinical Biomechanics, University of Southern Denmark, Odense, Denmark; 5 Department of Orthopedic Surgery, Aarhus University Hospital, Aarhus, Denmark; 6 Department of Public Health, Aarhus University, Aarhus, Denmark

**Keywords:** Occlusion training, Progressive resistance training, Muscle strength, Locomotion, Pain, Patients

## Abstract

**Objective:**

To compare the effect of low-load blood flow restricted resistance training (BFR-RT) versus high-load resistance training (HL-RT) on muscle strength, muscle mass, physical function, patient-reported outcomes, and adherence to training in clinical musculoskeletal populations.

**Data sources:**

Web of Science, Cochrane Central, Medline, Embase, SportDiscus was searched on the 30^th^ May 2022.

**Review methods:**

This study was conducted as a systematic review and meta-analysis. Randomized Controlled Trials (RCTs) were included if they (i) included patients, (ii) comprised of a BFR-RT intervention protocol and a group who performed HL-RT (≥ 70%1RM) for at least eight exercise sessions, and (iii) involved at least 1 exercise that targeted the lower limbs. The Cochrane Risk of Bias tool was used to evaluate the risk of bias. The meta-analyses were performed using a random effects model with an adjustment to the confidence interval.

**Results:**

Seven RCTs comprising 303 participants (BFR-RT: *n* = 151; HL-RT: *n* = 152) were identified. HL-RT and BFR-RT showed similar gains in dynamic (1-10RM) knee extensor strength and leg press strength, quadriceps cross sectional area, sit-to-stand performance, and patient reported pain and function. There was a moderate effect favoring BFR-RT for increasing maximal isometric knee extensor strength. The grading of certainty in evidence was low-to-very low for all outcome variables.

**Conclusion:**

This systematic review and meta-analysis extends our current knowledge about BFR-RT and HL-RT as equally effective exercise methods for inducing gains in maximal muscle strength in healthy populations, by now also comprising patients suffering from various clinical musculoskeletal conditions. The certainty in the estimates was low-to-very low, prompting the inclusion of future higher-quality trials.

**Trial registration:**

PROSPERO ID (CRD42022337173). Registered June 18th 2022.

## Introduction

Recent systematic reviews with meta-analysis have suggested that low-load resistance training (20–50% of one repetition maximum (RM)) combined with blood flow restriction to the exercising limb (low-load blood flow restricted resistance training: BFR-RT) and high-load resistance training (HL-RT, ≥ 70% 1RM) are equally effective in inducing gains in skeletal muscle mass in healthy populations ranging from young-to-old [1–3]. Therefore, BFR-RT has been suggested as a feasible exercise method in various clinical populations, where either fragile post-surgical conditions or the injury itself may restrict patients from exercising at higher muscle loading intensities [4, 5]. Loss of skeletal muscle mass and strength due to immobilization or general unloading is a well-known challenge among patient populations [6–8]. Further, loss of muscle mass and strength has been associated with declines in physical function [9] which, ultimately, could result in chronically reduced physical function [10]. Regaining habitual levels of muscle mass and strength after periods of bedrest or unloading may be challenging and, as a consequence, deficits in muscle strength often persist despite systematic post-injury rehabilitation efforts [11, 12]. Therefore, it is considered of strong relevance for patients to engage in exercise-based activities that preserve or promote skeletal muscle mass and mechanical muscle function (strength, power, rate of development: RFD) to countermeasure the negative impact of disease burden and disuse on muscle morphology, maximal muscle strength and function performance [13].

A number of clinical studies have reported comparable gains in both muscle mass and maximal muscle strength with BFR-RT vs. HL-RT in patients suffering from knee osteoarthritis (OA) [14], anterior cruciate ligament reconstruction [15], rheumatoid arthritis [16], and patellofemoral pain syndrome [17, 18]. A recent meta-analysis by Lixandrao et al. [1] revealed a superior effect of HL-RT compared to BFR-RT on evoking gains in maximal muscle strength, whereas a subsequent meta-analysis by Grønfeldt et al. [19] reported comparable gains in maximal muscle strength in response to BFR-RT vs. HL-RT. Despite the increasing application of BFR-RT in various patient populations [14–17, 20–25], the available data has not been summarized in a systematic review and meta-analysis to investigate if BFR-RT is equally effective compared to HL-RT of inducing gains in (i) maximal isometric and dynamic muscle strength, (ii) skeletal muscle mass, and (iii) physical function in clinical populations.

Therefore, the aim of this systematic review and meta-analysis was to evaluate the effect of BFR-RT vs. HL-RT on lower limb muscle strength and mass, objectively measured physical function, patient-reported outcomes (function and pain), and adherence to training in given patient populations with musculoskeletal conditions.

## Materials and methods

### Search strategy

The protocol for this systematic review was published online at the International Prospective Register of Systematic Reviews (PROSPERO: CRD42022337173). The systematic review was performed according to the PRISMA [26] guidelines. Original peer-reviewed articles were identified by searching the following electronic databases on May 30^th^ 2022: Web of Science, The Cochrane Central Register of Controlled Trials, Medline, Embase and SportDiscus. An updated search was conducted April 23rd 2023 where no new studies were identified. No restrictions were used in terms of publication language or publication year. Specific search terms are presented in Table 1.

**Table 1:** Search strategy

**"OR"**	**"AND"**	**"OR"**	**"AND"**	**"OR"**	**"AND"**	**"OR"**
"High intensity"[Text Word]		"Low load" [Text Word]		"Resistance training"[Mesh]		"Blood flow occlusion"[Text Word]
"High load"[Text Word]		"Low intensity" [Text Word]		Exercise[Mesh]		"Occluded blood flow"[Text Word]
"Heavy load"[Text Word]		"Body weight" [Text Word]		"Strength training"[Text Word]		"Vascular Occlusion"[Text Word]
Progressive[Text Word]				"Resistance training"[Text Word]		"Vascular restriction"[Text Word]
"Heavy weight"[Text Word]				Exercis*[Text Word]		"Blood flow restriction"[Text Word]
				"Weight training"[Text Word]		Occlusion[Text Word]

### Inclusion and exclusion criteria

Inclusion criteria comprised randomized controlled trials involving patients suffering from a condition or injury that requires conservative, medical or surgical treatment. Included trials needed to have comprised of a specific intervention involving at least one intervention group performing low-load (≤ 50% 1RM) BFR-RT and a group performing conventional high-load (≥ 70% RM) resistance training, performed for at least eight exercise sessions. At least one exercise was required to target the lower limbs, performed with free weights, in weight machines, with elastic band resistance, or with body weight exercises. Loading intensity (% 1-RM or number of reps to failure) had to be reported. Included studies had to report on at least one of the following post-intervention outcome parameters: Maximal isometric or isokinetic knee extensor strength, repetition maximum knee extensor strength, repetition maximum leg press strength, quadriceps cross-sectional area (CSA), sit-to-stand (STS) performance, maximal walking speed, patient-reported function ( i) function reported disease-specific questionnaires or ii) global questionnaires), patient-reported pain (i.e. i) pain reported in disease-specific questionnaires, ii) global questionnaires, iii) Numeric Ranking Scale (NRS) for worst pain), adherence to training, or the number of dropouts.

Trials were excluded if the publication language was not English. We did not set restrictions for publication date.

### Study selection and data extraction

Study inclusion was managed in Covidence (Veritas Health Innovation, Melbourne, Australia). A combination of two reviewers (SJ, SKB/MH) independently screened titles and abstracts to identify potentially eligible trials based on predetermined criteria. The full text of potentially eligible papers was retrieved and independently assessed by the same reviewers to determine eligibility. Any disagreements were resolved via consensus or by consulting a fourth author (IM) when necessary. A combination of two reviewers (SJ, MH/MBB) separately performed data extraction using a pre-specified excel spreadsheet. Disagreements were solved by discussion until agreement was reached. Otherwise, a third author was consulted (IM). The following data were extracted from each study:

1. Trial characteristics (sample size, first author name, year of publication, type of trial, country).
2. Participant characteristics (age, sex, body mass).
3. Intervention procedures for each group, including exercise protocols.
4. Co-interventions, if any, reported for each group.
5. Outcomes variables reported, including time of assessment.

## Quality assessment

### Risk of bias assessment

Two reviewers (SJ, IM) independently assessed the risk of bias using Cochrane’s risk of bias tool version 2.0 (RoB) [27] and discrepancies were resolved through discussion until reaching consensus. RoB assessment scores on the reporting of judgement items were: (i) Adequate (*bias, if present, is unlikely to alter the results seriously),* (ii) Unclear *(a risk of bias that raises some doubt about the results)*, and (iii) Inadequate (*bias may alter the results seriously)*, corresponding with (i) Low risk, (ii), Some concerns, and (iii) High risk of bias. The RoB analysis was performed separately for objective outcomes (i.e. lower limb strength, quadriceps CSA, STS) and patient-reported outcomes (function and pain) and included five distinct aspects of reporting: the randomization process, deviations from the intended intervention, missing outcome data, measurement of the outcome variables, and selected reporting of the obtained results.

### Certainty assessment

Two reviewers (SJ, IM) rated the certainty in the evidence for each outcome variable using Grades of Recommendation, Assessment, Development, and Evaluation (GRADE) [28, 29] (Table 3). Overall GRADE scores were categorized as “very low”, “low”, “moderate”, or “high” [29].

### Statistical analyses

Outcome variables were reported using different units across studies. Hence, the effects of low-load BFR-RT and HL-RT were evaluated by calculating the post-intervention standardized mean difference (SMD) estimated by Hedges’ g as $\frac{mean1 - mean2}{SDpooled}$ along with the 95% confidence interval (CI), where mean1 denotes the post-intervention score for BFR-RT group and mean2 denotes the post-intervention score for the HL-RT group. Also, we included the 95% prediction interval (PI) graphically in the figures. For interpretation of SMD, the following definitions were adopted: > 0.2 small effect; > 0.5 moderate effect; > 0.8 large effect [30]. SD^*^_pooled_.

Heterogeneity between included studies was assessed using the *I*^*2*^ statistics and interpreted as low (*I*^*2*^ = 30–60%) and high (*I*^*2*^ ≥ 60%) [31, 32]. Given that a relatively small number of trials were included in each meta-analysis (often less than 6 studies), a random effects model was performed with an adjustment to the CI as proposed by Sidik and Jonkman [33].

All statistical analyses were conducted using Stata 17.0 (StataCorp, TX, USA).

## Results

### Study selection

**Fig. 1:**
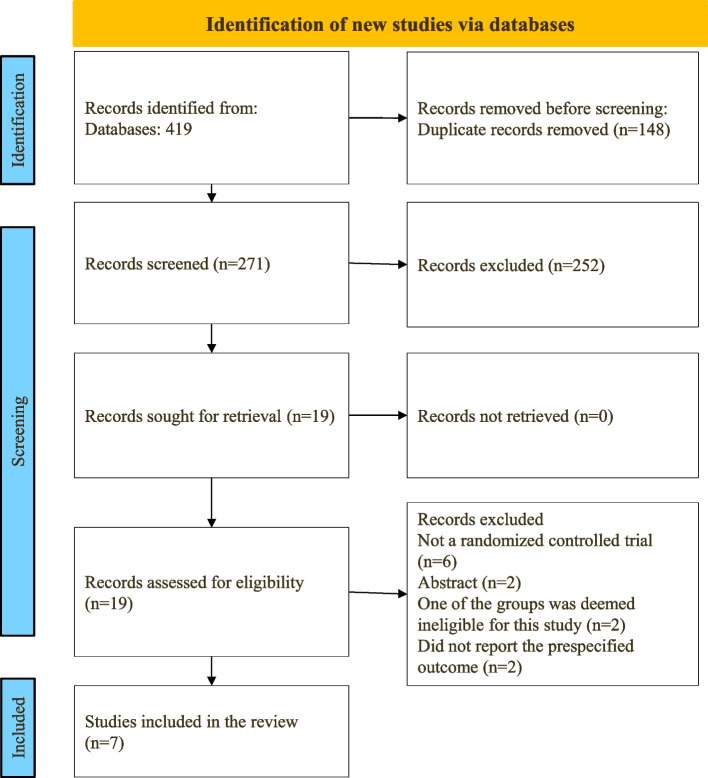
PRISMA flow chart

A total of 419 study records were identified, of which 148 were discarded as duplicates (Fig. 1). From the remaining 270 studies, 251 were excluded through title screening and abstract assessment, while 19 studies were excluded following full-text reading, and one study was excluded as SMD could not be calculated from the reported data [34]. Consequently, a total of seven studies [14–18, 35, 36] were included in the present meta-analysis.

### Study characteristics

Individual trial characteristics are summarized in Table 2. A total of 303 patients allocated to either BFR-RT or HL-RT were included in the overall meta-analysis (152 BFR-RT/151 HL-RT). Mean age in each individual trial ranged from 25.5 ± 10.4 to 63.3 ± 7.0 years, altogether comprising 61% patients who were women. The included study populations were: patellofemoral pain syndrome [17, 18], knee OA [14, 36], anterior cruciate ligament reconstruction surgery [15], rheumatoid arthritis [16], and military personnel suffering from musculoskeletal lower-limb injuries [35].

**Table 2:** Study characteristics

**Study** Diagnosis	**BFR RT** N (m/f) Age	Exercises	Intensity Mean LOP Cuff width	Sets Reps Rest between sets Frequency Duration	Adherence Dropouts Adverse events Supervision	**Outcomes reported**
	**HI RT**					
Bryk 2016 [28] Knee OA	17 (17/0) 62.3 ± 7.0	Knee extension (machine, 90°-0°)	30% 1RM 200 mmHg N/I	3 set, 30 N/I 3/week 6 weeks	N/I 0 0 Yes	Isometric knee extension, Numeric Pain Rating Scale
	17 (17/0) 60.4 ± 6.7	Knee extension (machine, 90°-0°)	70% 1RM None None	3 10 N/I 3/week 6 weeks	N/I 0 0 Yes	
Constantinou 2022 [10] PFP	30 (17/13) 25.5 ± 14	Hip extension (machine) Hip abduction (machine) Knee extension, unilateral (machine, 90°-45°) Leg press, unilateral (machine, 0°-45°)	30% 1RM 70% LOP 10 Cm	4 30–15-15–15 30 s 3/week 4 weeks	83% 0 0 Yes	Isometric knee extension
	30 (16/14) 30.5 ± 16	Hip abduction, (ankle weights, side-lying) Hip extension (machine) Hip abduction (elastic band, standing) Hip external rotation (elastic band, sitting) Knee extension (machine, 90°-45°) Leg press, unilateral (machine, 0°-45°)	70% 1RM None None	3 10 120 s 3/week 4 weeks	83% 0 0 Yes	
Ferraz 2018 [6] Knee OA	16 (0/16) 60.3 ± 3	Leg press, bilateral (machine) Knee extension, bilateral (machine)	20–30% 1RM 70% LOP 175 mm	4–5 15 60 s 2/week 12 weeks	90% 4 0 Yes	Knee extension strength (RM), Leg press strength (RM), Sit-to-stand, WOMAC Physical function, WOMAC pain
	16 (0/16) 59.9 ± 4	Leg press, bilateral (machine) Knee extension, bilateral (machine)	50–80% 1RM None None	4–5 10 60 s 2/week 12 weeks Yes	91% 6 4 Yes	
Giles 2017 [9] PFP	40 (16/24) 28.5 ± 5.2	Leg press (0°-60) Knee extension ((90°-45°)	30% 1RM 60% LOP N/I N/I	4 30–15-15–15 30 s 3/week 8 weeks	83% 5 0 Yes	Isometric knee extension strength, VAS worst pain in the last week
	39 (20/19) 26.7 ± 5.5	Leg press (0°-60) Knee extension (90°-45°)	70% 1RM 0 mmHg 5 cm	3 7–10 N/I 3/week 8 weeks	80% 5 0 Yes	
Hughes 2019 [7] ACLR	14 (7/5) 29 ± 7	Leg press, unilateral (0°-90°) Knee extension,, unilateral (0°-90°)	30% 1RM 80% LOP 11.5 cm	4 30–15-15–15 30 s 2/week 8 weeks	91.2% 2 0 Yes	Isometric knee extension strength, Leg press strength (RM), patient-reported pain
	14 (10/2) 29 ± 7	Leg press, unilateral (0°-90°) Knee extension, unilateral (0°-90°)	70% 1RM None None	3 10 30 s 2/week 8 weeks	87.5% 2 0 Yes	
Ladlow 2018 [27] Military Personal suffering from orthopedic lower limb injuries	14 (14/0) 33 ± 6	Leg press, bilateral (machine) Knee extension, bilateral (machine)	30% 1RM 60% LOP 10 cm	4 30–15-15–15 30 s 2/day 3 weeks	100% 0 0 Yes	Knee extension strength (RM), Leg press strength (RM)
	14 (14/0) 28 ± 7	Deadlift Back squat Lunges	6-8RM None None	4 6–8 180 s 3/week 3 weeks	90% 0 0 Yes	
Rodrigues 2020 [8] Rheumatoid Arthritis	16 (0/16) 59.6 ± 3.9	Bilateral leg press Bilateral knee extension	20- 30%1RM 70% LOP 175 mm	4–5 15 60 s 2/week 12 weeks	86.2% 0 0 Yes	Leg press strength (RM), Sit-to-stand
	16 (0/16) 58.0 ± 6.6	Bilateral leg press Bilateral knee extension	50–80% 1RM None None	4–5 10 60 s 2/week 12 weeks	86.6% 1 1 Yes	

All trials included at least one intervention group performing BFR-RT and at least one intervention group performing HL-RT, with six trials reporting the intensity as %1RM [14–18, 36] and one trial reporting intensity as 8RM [35]. Duration of BFR-RT varied from 2–3 sessions/week for 4–12 weeks in six of the included trials [14–18, 36] and 2 sessions/day for three weeks in Ladlow et al. [35]. Duration of HL-RT varied from 2–3 sessions/week for three to 12 weeks in all trials. Adherence to training ranged from 83%-100% and 83%-90% for BFR RT and HL-RT, respectively. Ferraz et al. [14] reported 10 dropouts (BFR-RT: *n* = 4 vs. HL-RT: *n* = 6) and four adverse events (HL-RT: *n* = 4), Giles et al. [17] reported 10 dropouts (five in each group), Hughes et al. [15] reported four dropouts (two each group), and Rodrigues et al. [16] reported 1 drop out (HL-RT: *n* = 1) and one adverse event (HL-RT: *n* = 1).

### Risk of bias assessment

**Fig. 2:**
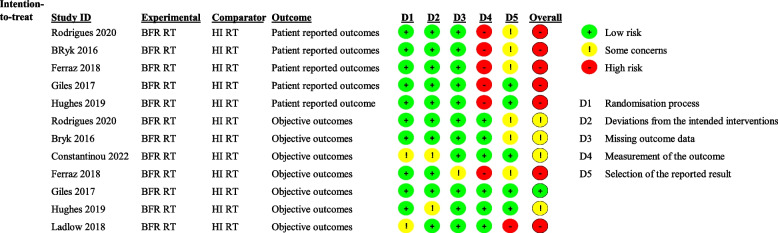
Risk of bias assessment

Our RoB assessment for all included trials is presented in Fig. 2. RoB was deemed low for the objective outcome measures reported in Giles et al. [17], while some concerns were found for the objective outcome measures reported by Rodrigues et al. [16], Bryk et al. [36], Constantinou et al. [18], and Hughes et al. [15]. High risk of bias was noted for Ferraz et al. [14] and Ladlow et al. [35] for their objective outcome measures. RoB for the patient reported outcome variables was deemed to be high in all included trials.

### Certainty in evidence

The grading of certainty in evidence was low-to-very low for all outcome variables (Table 3).

**Table 3:** Summary of findings (SoF) table

**Certainty assessment**							**Summary of findings**				
**№ of studies**	**Study design**	**Risk of bias**	**Inconsistency**	**Indirectness**	**Imprecision**	**Publication bias**	**№ of patients**		**Effect**	**Quality of evidence**	
							**Intervention**	**Control**	**Absolute [95% CI]**		
**Isometric knee extension, follow up**											
4	RCT	1 serious	No serious	No serious	1 serious	No serious	101	100	0.47 [ 0.12, 0.83]	Low ⨁⨁□□	^a,e^
**Knee extension strength (RM), follow-up**											
3	RCT	No serious	1 serious	No serious	1 serious	No serious	46	46	-0.08 [-0.71, 0.54]	Low ⨁⨁□□	^c,e^
**Leg press strength (RM), follow-up**											
4	RCT	1 serious	1 serious	No serious	1 serious	No serious	46	46	-0.17 [-0.97, 0.63]	Very low ⨁□□□	^a,c,e^
**Sit-to-stand, follow-up**											
2	RCT	1 serious	No serious	No serious	1 serious	No serious	60	60	-0.02 [-0.76, 0.71]	Low ⨁⨁□□	^a,e^
**Quadriceps, cross sectional area**											
4	RCT	1 serious	No serious	No serious	1 serious	No serious	86	85	-0.10 [-0.42, 0.31]	Low ⨁□□□	^c,d^
**Patient-reported Pain, follow-up**											
2	RCT	2 serious	No serious	No serious	2 serious	No serious	103	103	0.61 [-0.90, 2.12]	Very low ⨁□□□	^a,b,d,e^
**Patient-reported Function, follow-up**											
4	RCT	2 serious	1 serious	No serious	2 serious	No serious	32	32	-0.13 [-0.62, 0.36]	Very low ⨁□□□	^a,b,d,e^

*CI* Confidence interval, *ES* Effect size, *SMD* Standardized mean difference

^a^ Failure to follow intention-to-treat principles in all studies

^b^ Lack of blinding

^c^ Differences in populations

^d^ Differences in assessment methods

^e^ Small study population

### Synthesis of results

**Fig. 3:**
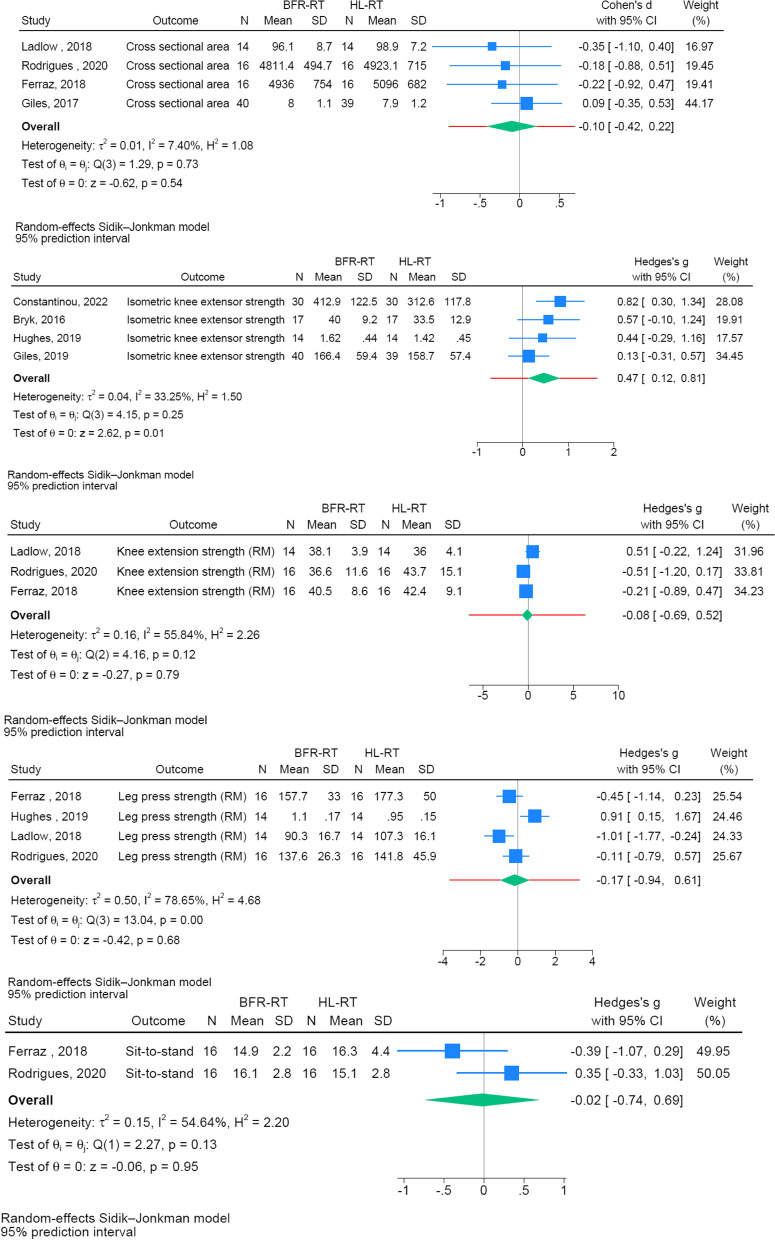
Forrest plots on muscle mass, muscle strength, and sit-to-stand performance

**Fig. 4:**
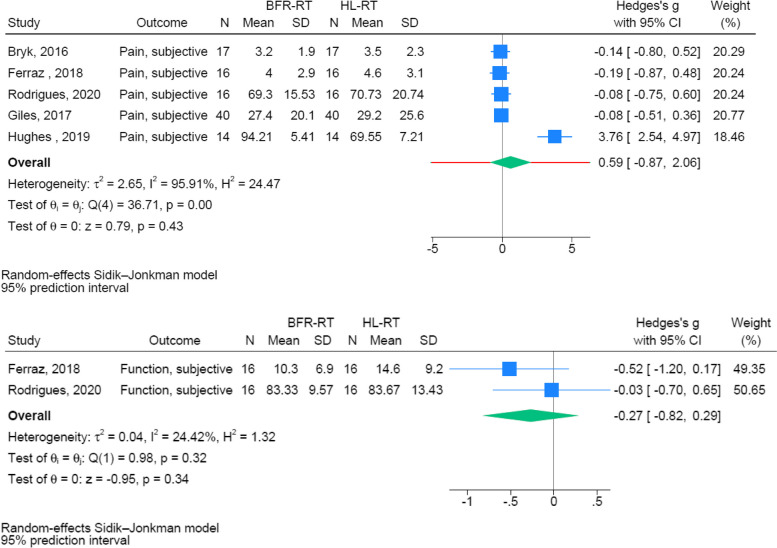
Forrest plots on post-intervention patient-reported outcomes

Seven RCTs were included in the present meta-analyses [14–18, 35, 36]. A number of separate meta-analyses were performed to compare the intervention effect of BFR-RT vs. HL-RT on: knee extensor MVC [15, 17, 18, 36], Maximal (1–10 RM) dynamic knee extensor strength (knee extensor strength) [14, 16, 35], maximal (1–10 RM) dynamic leg press strength (leg press strength) [14–16, 35], quadriceps muscle CSA [14, 16, 17, 35], STS performance [14, 16], (Fig. 3), as well as on patient reported function [14] and patient reported pain [14, 15, 17, 36] (Fig. 4).

No post-intervention differences were observed between BFR-RT and HL-RT on knee extensor strength, leg press strength, quadriceps CSA, STS performance, patient reported function, or patient reported pain (Fig. 3). In contrast, a small effect favoring BFR-RT was observed for knee extensor MVC (SMD = 0.47 [0.12,0.81]) (Fig. 3).

## Discussion

The main finding in the present study was that BFR-RT and HL-RT produced comparable follow-up outcomes for dynamic lower limb muscle strength, knee extensor muscle CSA, STS performance, and patient reported function and pain in patient groups involving patellofemoral pain syndrome [17, 18], knee osteoarthritis [14, 36], anterior cruciate ligament reconstruction surgery [15], rheumatoid arthritis [16]; and musculoskeletal lower-limb overuse injury [35]. Interestingly, while training adherence and dropout rates were equal between BFR-RT and HL-RT, fewer adverse events were noted with BFR-RT (0 vs. 5 adverse events). Notably also, larger follow-up scores in knee extensor MVC were observed in patients randomized to BFR-RT than HL-RT intervention (Fig. 3). Consequently, low-load BFR-RT may be considered a viable modality with no evidence of difference in follow-up scores between BFR-RT and HL-RT in maximal muscle strength, muscle mass, physical function and patient reported outcomes across various musculoskeletal and rheumatoid patient populations [14–18, 35, 36].

### Maximal muscle strength

Notably, significantly higher follow-up scores in knee extensor MVC were observed in response to BFR-RT compared to conventional HL-RT. This observation may appear surprising given that all the individual studies measuring knee extensor MVC [15, 17, 18, 36] were unable to detect any between-group difference in the magnitude of change, and previous meta-analyses have reported either comparable gains in maximal muscle strength with BFR-RT vs. HL-RT [19] or larger gains with HL-RT [1]. However, as proposed in two previous studies [37, 38] an increase in unspecific strength (i.e. a task none-similar to the exercises performed) is more difficult to detect. Based on the 95%PI our results appear to conform with these previous results [37, 38]. Although interestingly, a sub-group analysis conducted by Giles et al. [17] showed greater improvements in maximal knee extensor MVC following BRT-RT vs. HL-RT in patients with patellofemoral pain syndrome affected by pain when exercising. This may suggest that if musculoskeletal pain is limiting the ability to perform resistance exercise, which may especially be the case during the early phase of rehabilitation, reducing the magnitude of mechanical strain on the affected limb using low exercise loads (20–30% 1RM) and applying ischemia during and between the exercise bouts may have increased the tolerance towards and thereby effectiveness of the training performed.

### Maximal dynamic muscle strength

In terms of maximal leg press- and knee extensor strength, no differences were observed between follow-up scores for BFR-RT and HL-RT. However, as illustrated in Table 2, both BFR-RT and HL-RT appear to induce significant gains in strength from baseline to follow-up. Therefore, the results from the present meta-analyses appear to be consistent with the individual study findings since all studies included in the meta-analysis found comparable strength gains in leg press strength and dynamic knee extensor strength following BFR-RT vs. HL-RT (Table 2) [14–16, 35]. These observations support previous conclusions, suggesting that adaptations to strength usually is greater in the exercises that was trained (specific strength) [19, 37]. The present meta-analysis demonstrate similar trend in patients suffering from various lower limb conditions.

To achieve gains in skeletal muscle size and strength, it is imperative to engage fast-twitch type II muscle fibers as these fibers generally demonstrate a more pronounced hypertrophic capacity compared to the slow-twitch type I muscle fibers [39, 40]. Notably, both HL-RT and low-load BFR-RT appear to mediate gains in muscle strength and size, respectively, with no evidence of differences in follow-up scores in selected patient groups (present data) as well as in healthy populations which has been demonstrated in previous systematic reviews [1, 19, 41].

### Muscle cross-sectional area

Preserving skeletal muscle mass is of vital importance in a number of patient populations [3, 13, 42]. However, due to post-surgical load restriction guidelines and/or pain restrictions [5, 42, 43], it can often be difficult to employ sufficiently high loading intensities to promote skeletal muscle hypertrophy in given individual patients. Therefore, BFR-RT has become increasingly popular in the rehabilitation of musculoskeletal disorders as its stimulating effects on muscle growth has become well-established [3, 19, 44]. In accordance with Henneman’s ‘size principle’ heavy training loads normally are required to achieve maximal muscle fiber recruitment within the exercising muscle, which is a prerequisite for evoking adaptive changes in muscle morphology and neural activation [45, 46]. With low-load BFR resistance exercise, the resulting ischemic intramuscular environment give rise to metabolic stress mediators that have been suggested to increase type II muscle fiber recruitment, induce muscle cell swelling resulting in increased mechanotransducive signaling, and to stimulate satellite cell proliferation and myonuclei accretion [39, 47], altogether contributing to the hypertrophic response. The present observation indicating no evidence of difference in follow-up scores of BFR-RT and HL-RT in quadriceps muscle CSA in clinical patient groups comprising patellofemoral pain syndrome [17, 18], musculoskeletal lower-limb overuse injury [35], rheumatoid arthritis [16], and knee osteoarthritis [14, 36] (cf. Fig. 3) may not be surprising. In line with our study, although comprising a fewer number of studies, similar observations were reported in a recent meta-analysis involving patients with osteoarthritis and rheumatoid arthritis Thus, as suggested by Ladlow et al. [35], exercising at lower loading intensities reduces the joint forces hence reducing the degree of joint/injury-specific pain and ultimately allowing to reach higher levels of perceived exertion compared to HL-RT. Also, reducing the load can increase the overall greater training volume, which have been proven to equally efficient in increasing skeletal muscle CSA as HL-RT at 60–80% 1RM [48].

### Physical function

Only Ferraz et al. [14] and Rodrigues et al. [16] assessed STS performance after BFR-RT vs. HL-RT. STS function is commonly used test to assess physical function, especially in older patient populations and patients suffering from lower limb OA [25, 49, 50]. As indicated by the present meta-analysis no significant difference in follow-up scores emerged between the groups engaging in BFR-RT compared to HL-RT. As all four groups displayed a significant within-group improvement from baseline-to-follow-up (Table 2), both BFR-RT and HL-RT appeared able to induce changes in physical function (Fig. 3).

### Patient-reported outcomes for pain and function

While improved following training (Table 2), none of the patient-reported outcome variables were selectively favored by BFR-RT or HL-RT. Interestingly, this observation is somewhat inconsistent with the findings of the individual studies. Thus, Hughes et al. [15] found significantly greater improvements in measures of patient-reported physical function and pain over eight weeks of training with BFR-RT vs HL-RT. In addition, Ferraz et al. [14] noted that BFR-RT and HL-RT led to similar improvements in patient-reported physical function, while only BFR-RT improved pain. Contributing to the contradictory results, Rodrigues et al. [16] reported that only HL-RT improved patient-reported physical function while BFR-RT improved pain. Nonetheless, the present meta-analysis on selected patient-reported outcome found no evidence of difference in follow-up scores between on these parameters.

### Adherence and adverse events

Collectively, a high adherence (80–100%) to the prescribed training was observed across studies for both intervention modalities. Further, a low number of dropouts were observed, with only Ferraz et al. [14] reporting a relatively high dropout rate (10 of 32 participants). Also, four of a total of five reported adverse events across trials were observed by Ferraz et al. [14], all caused by exercise-induced knee pain with HL-RT. A single adverse event was reported by Rodrigues et al. [16], which was due to exercise-induced patellofemoral pain with HL-RT. Notably, no adverse events were reported with BFR-RT across a variety of patient populations. Thus, based on the present observations of equal follow-up scores in muscle strength, muscle mass, physical function (STS), and patient-reported outcomes along with high adherence and no (BFR-RT) or only few (HL-RT) adverse events, patient preferences and motivation should be taken into account, when deciding on whether to apply BFR-RT or HL-RT in the rehabilitation setting. However, it is important to recognize that BFR-RT protocols are typically cautiously applied in clinical trials, resulting in relatively strict in- and exclusion criteria. Thus, the low number of adverse events observed in the trials included in the present meta-analysis may not necessarily reflect a general safety profile of BFR-RT, as more fragile patients often are selectively excluded from longitudinal exercise studies. Nonetheless, the present observations along with previous study reports suggest that high training adherence and ample safety precautions can be achieved with the use of BFR-RT in selected clinical populations [21–23, 51, 52].

#### Methodological considerations

In terms of methodological strengths, the present study conformed to guidelines outlined by the Cochrane Handbook for Systematic Reviews of Interventions [version 6.2 (updated February 2021)], the PRISMA statement [26] and the GRADE Evidence to Decision framework [53]. Specifically, all inclusion and exclusion criteria were stated a priori, while all included trials used a RCT design and reported data on key exercise variables (i.e. intensity, type, frequency and duration).

A number of limitations may be mentioned with the present meta-analysis. First, the relatively low number of studies (*n* = 7, 303 patients) included in the present analysis along with relatively small populations in the individual studies limits the interpretation of the present observations. This is also reflected by large PIs for all outcome parameters, suggesting that future studies may impact the results of the present study. However, since only RCTs were included and we applied a random effects analysis model adjusted for CI due to the low number of studies included [33, 54], the present results may still represent a valid assessment of the follow-up scores between BFR-RT versus HL-RT in the rehabilitation of selected patient groups.

Basic exercise parameters such as training frequency, duration, load, and the total training volume for the lower limbs (specifically the quadriceps muscle) varied markedly between the included studies. For instance, in Ladlow et al. [35] the BFR-RT group trained twice daily for three weeks while the HL-RT Group performed three HL-RT per week for three weeks. However, it was beyond the scope of this systematic review to investigate the specific dose–response relationship of BFR-RT versus HL-RT. Also, we allowed studies to be included with as little as eight planned exercise sessions. Obviously, eight sessions would result in a very low total training volume, however, to ensure inclusion of all studies comparing BFR-RT and HL-RT, we decided on this cut-off point.

Initially, we intended to also include patients suffering from various cardiovascular and medical conditions (cf. PROSPERO registration) to allow sub-group analysis on selected outcome parameters. However, no eligible trials on patients suffering cardiovascular diseases were retrieved. Interestingly, from our title/abstract screening, we excluded several protocols registered on clinicaltrial.org on effect of BFR-RT in patients suffering from chronic obstructive pulmonary disease, type 2 diabetes, coronary heart disease patients, chronic heart failure patients, and patients with ischemic stroke. This warrants an update of the current systematic review and meta-analyses within a few years.

Since the present meta-analysis included patients with a wide diagnosis range, considerable inter-individual variations were observed in terms of basic patient characteristics such as age, body mass, body mass index, and baseline measures of strength and physical activity. Consequently, the present study populations were quite inhomogeneous. Conversely, the objective outcome measures evaluated in the present meta-analysis (e.g. maximal isometric and dynamic muscle strength, muscle CSA, STS performance) were obtained using validated assessment methods usually considered of high reliability. Given that the present meta-analysis evaluated follow-up scores between BFR-RT versus HL-RT on a number of clinically important outcome variables, we believe that the present observations and conclusions may aid clinical decision making in the prescription of exercise-based rehabilitation in given patient populations.

The present study did not compare the interventions to a non-exercising control group. Therefore, we cannot draw any conclusions on effectiveness of the BFR-RT and HL-RT. Also, none of the trials in the meta-analyses included a non-exercising control group. Ferraz et al. [14] and Rodrigues et al. [16] included a group performing low-load resistance training (LL-RT) without BFR. Both studies demonstrated that LL-RT was inferior to BFR-RT og HL-RT in inducing gains in strength, physical function [14, 16]. In contrast, LL-RT appeared able to induce significant within-group changes in pain and patient-reported physical function [14]. Thus, to determine the effectiveness of BFR-RT and HL-RT, future studies are warranted to include non-exercising controls.

Notably, the certainty in the estimates was deemed low-to-very low in the present meta-analyses, mainly resulting from a failure to adopt the intention-to-treat principle, lack of observer/tester blinding, inhomogeneous populations, differences in assessment methods, and small study populations. This means that the outcome of present meta-analysis may change with the inclusion of future high-quality trials.

## Conclusions

Based on the present meta-analysis, the current evidence shows that BFR-RT and HL-RT produce comparable follow-up scores in maximal muscle strength, quadriceps cross-sectional area, physical function, and patient reported outcome measures of function and pain. BFR-RT and HL-RT resulted in similar exercise adherence rates, and involved only few and minor adverse events. BFR-RT may be considered a feasible exercise method in the clinical rehabilitation setting. Certainty in the derived estimates was low-to-very low, prompting for future high-quality trials, including non-exercising control groups.

## Data Availability

Data sharing is not applicable to this article as no datasets were generated or analysed during the current study.
